# Sarcopenia: how to measure, when and why

**DOI:** 10.1007/s11547-022-01450-3

**Published:** 2022-01-18

**Authors:** Alberto Stefano Tagliafico, Bianca Bignotti, Lorenzo Torri, Federica Rossi

**Affiliations:** 1 Department of Health Sciences (DISSAL), University of Genoa, Genova, Italy; 2 Department of Radiology, IRCCS Policlinico San Martino Hospital, Genova, Italy; 3 Department of Surgery, University of Pisa, Pisa, Italy; 4 Department of Radiology, Santa Corona Hospital, Pietra Ligure, Italy

**Keywords:** Sarcopenia, Imaging, Muscle mass, Radiology, Computed tomography, Ultrasound, Magnetic resonance

## Abstract

Sarcopenia indicates a loss of skeletal muscle mass, a condition that leads to a decline in physical performance. In 2018, the European Working Group on Sarcopenia in Older People met to update the original definition of sarcopenia: New scientific and clinical insights were introduced to emphasize the importance of muscle strength loss as a prime indicator of probable sarcopenia. In addition, the skeletal muscle is not only the organ related to mobility, but it is recognized as a secondary secretory organ too, with endocrine functions influencing several systems and preserving health. In this perspective, radiology could have a major role in early detection of sarcopenia and guarantee improvement in its treatment in clinical practice. We present here an update of clinical knowledge about sarcopenia and advantages and limitations of radiological evaluation of sarcopenia focusing on major body composition imaging modalities such as dual-energy X-ray absorptiometry, CT, and MRI. In addition, we discuss controversial such as the lack of consensus or standardization, different measurement methods, and diagnostic radiological cutoff points. Sarcopenia evaluation with radiological methods could enhance the role of radiologist in performing studies with relevant impact on medical and social outcome, placing radiology at the pinnacle of quality in evidence-based practice with high-level studies.

## Definition of Sarcopenia

Sarcopenia is defined as an age-associated loss of skeletal muscle function and muscle mass occurring in approximately 6–22% of older adults [1]. Sarcopenia is identified by several criteria according to European consensus [2] taking into account for low muscle strength, low muscle quantity or quality and low physical performance. Low muscle strength is related to probable sarcopenia, diagnosis is confirmed when there is also low muscle quantity or quality, whereas sarcopenia is severe when low physical performance is present [2].

Several pathological conditions can lead to sarcopenia even in subjects younger than 65 years old. Sarcopenia assessment methods were therefore needed to evaluate both muscle mass and function.

Although clinically available grip strength and gait speed tests remain the simplest and cheapest methods, muscle mass can be estimated in several ways, most of them including radiological techniques. Even if in 2021, bioelectric impedance analysis is still a widely used and easily available method in clinical practice [2], it has several disadvantages, especially in de- or hypo-hydration status. Indeed, some differences in intracellular and transcellular penetrations between genders and in individuals reduce the consistency and accuracy of this technique [3]. In addition, bioelectric impedance analysis can determine fat-free mass and total body water but not muscle function or structure and it is more reliable in patients without significant fluid and electrolyte. Various tests and tools can be used to characterize sarcopenia in clinical practice, and appropriate choice may depend on several factors: patient-related (disability, mobility), technical-related (availability of tests) or disease-related (need to monitor progression of disease, rehabilitation and recovery) [1].

## Clinical relevance of Sarcopenia and the endocrine function of the skeletal muscle

**Fig. 1:**
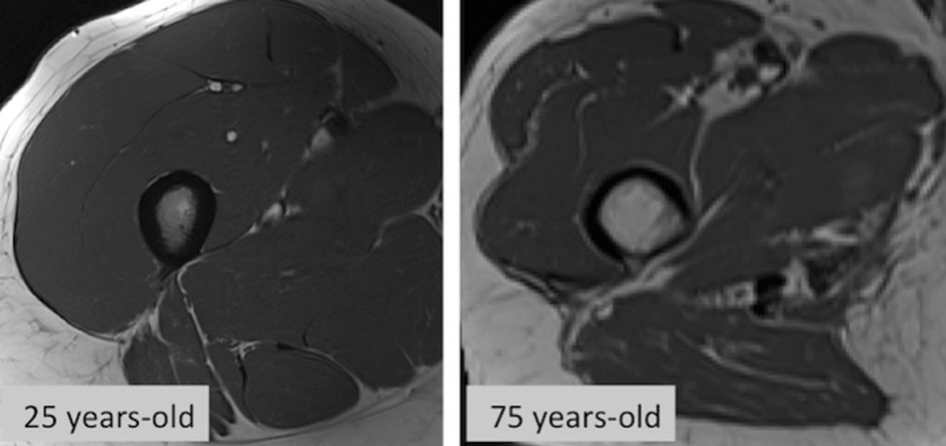
Proportion of lean body mass versus fat in the thighs of a young versus old woman as normal aging process. See text for detail

Sarcopenia is a term of Greek derivation consisting of two words: flesh (*sarko*-, compositional element from *sárks sarkós, σαρκο*) and loss, poverty (penia, πενία). Indeed, sarcopenia indicates a loss of skeletal muscle mass, a condition that leads to a decline in physical performance [1]. Historically, this term was first introduced in September 1994 at the workshop of the National Institute on Aging. Sarcopenia is an age-related phenomenon characterized by a dramatic decline in lean body mass over the decades of life (Fig. 1) [1]. In 1977, a study evaluated the correlation between creatinine excretion, as a measure of muscle mass, and the decline in basal metabolic rate, especially after the age of 40 [2]. The results of this study highlighted how the loss of skeletal muscle mass was related to both reversible and irreversible phenomena [2]. In addition, the age-related sarcopenia was included in the International Classification of Diseases (ICD-10-CM) with the code M62.84. Although in 2021 there are at least three major consensus groups publishing about sarcopenia (the European Working Group on Sarcopenia in Older People, EWGSOP, the European Society for Clinical Nutrition and Metabolism Special Interest Group, ESPEN-SIG, and the International Working Group on Sarcopenia, IWGS), there is still a lack of worldwide agreement right on the definition of sarcopenia [3–5]. Probably, the most widely accepted and used definition of sarcopenia is from the European Working Group (EWGSOP, the Sarcopenia Working Group) encompassing both the presence of low muscle mass and low muscle function (strength and performance) [3]. In 2018, the European Working Group on Sarcopenia in Older People met again (EWGSOP2) to update the original definition of sarcopenia introducing new scientific and clinical insights developed in the last 10 years [4]. These updates emphasized the importance of muscle strength loss as a prime indicator of probable sarcopenia. In addition, it emerged that sarcopenia could have both acute and chronic course. Recommendation on the use of algorithms for case-finding and specific cutoff points for sarcopenia assessment, necessarily followed [6]. The final goal is to facilitate early detection and to guarantee improved treatment of sarcopenia. In clinical practice, there is still today a sort of overlap between the term “frailty” and “cachexia.” Sarcopenia usually precedes frailty, a more complex syndrome, which by definition should meet three of these five criteria: low grip strength, low energy, slowed walking speed, low physical activity and unintentional weight loss [5]. Cachexia, on the other hand, is a kind of secondary sarcopenia normally caused by both weight loss and malnutrition in severe chronic diseases such as cancer, heart failure, kidney disease and obstructive pulmonary disease [6]. From a practical point of view, sarcopenia could be defined as a progressive and global skeletal muscle condition related to an increased likelihood of adverse events such as falls, fractures, physical frailty and even mortality. It has been evaluated that sarcopenia could be highly prevalent in 29–33% in community-dwelling populations and long-term care individuals [7]. The main reason for the development of primary sarcopenia is that aging itself determines worsening of muscle fibers and even α-motor neurons probably due to a reduced production of sex steroid hormones and growth hormone [6]. Secondary sarcopenia could be determined by malnutrition, inactivity, drugs and certain medical treatments.

**Fig. 2:**
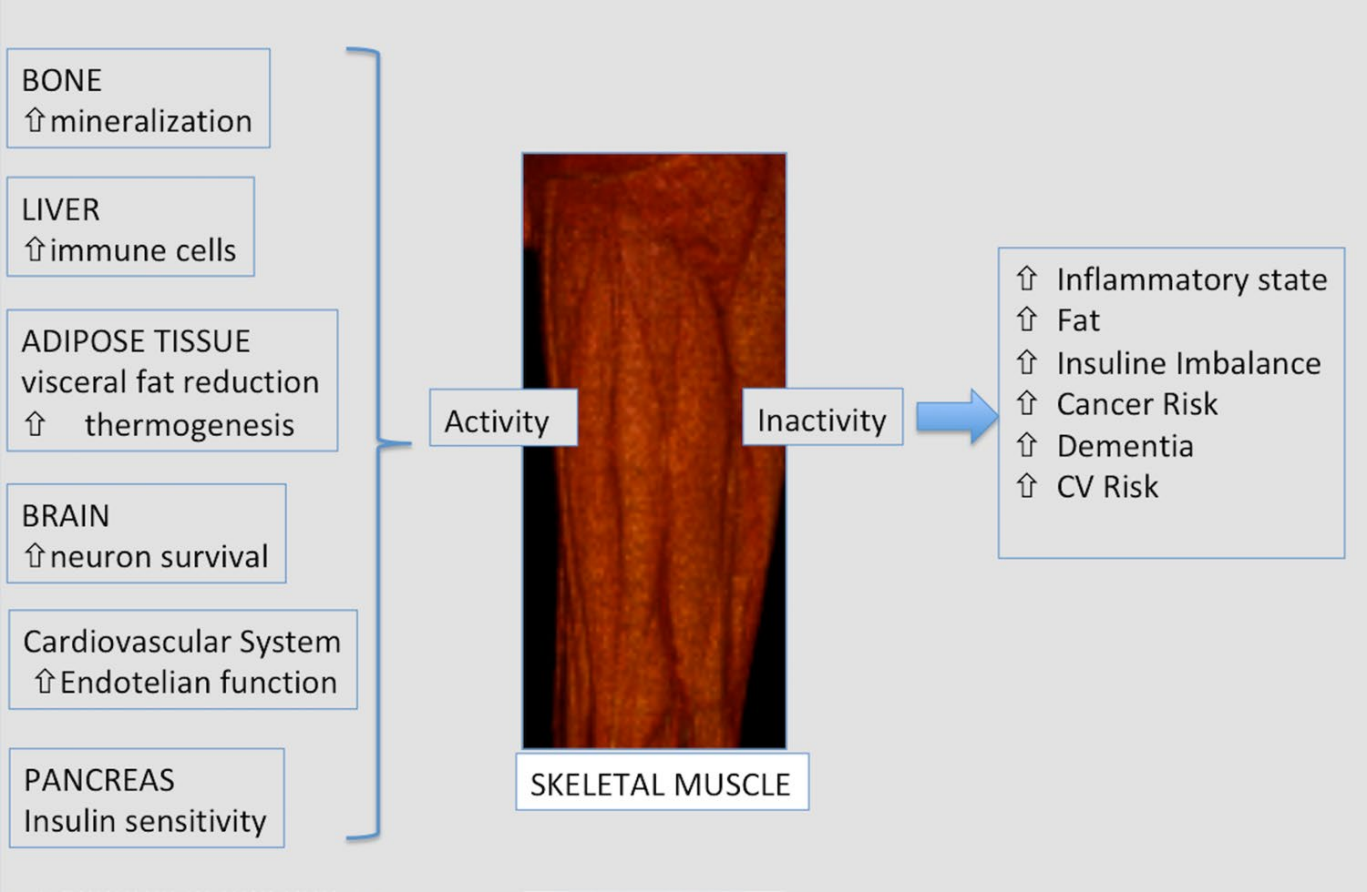
Muscle as secretory organ. Muscle contraction stimulates the production of different myokines. Myokines act as an endocrine stimulus on distant organs, partly explaining the known association between sedentary life and several chronic diseases

The skeletal muscle is not only the organ related to mobility, which is simply able to contract if properly stimulated, but also, recent physiological studies recognized it as a secondary secretory organ with endocrine functions via the myokines system. Myokines are cytokines such as interleukine-6 and brain-derived neurotrophic factor (BDNF) influencing glucose liver metabolism, lipolysis in the adipose tissue, pancreatic beta-cell and neuron vitality [6]. After muscular contraction, the myokine production is important to prevent the development of a proinflammatory status and metabolic imbalance starting sarcopenia and fat accumulation [8–10] (Fig. 2). In other words, in people with a sedentary lifestyle, there is a nutrient overload resulting in accumulation of fat and consequent disorder in adipocyte metabolism. Indeed, imbalanced adipocyte metabolism determines secretion of adipokines, which are primarily proinflammatory cytokines. These proinflammatory cytokines, on the contrary, are inhibited by the myokines, released during skeletal muscle contraction [13]. Consequently, the balance between adipokines and myokines regulates metabolic homeostasis and prevents not only metabolic diseases but also represents an effective and under-evaluated cross talk between skeletal muscle and other target organs, such as the adipose tissue, the bone, the liver, the kidney and even the brain [3]. In the future, there may be developments in myokine research as a drug candidate to explore novel paths for the elaboration of applicable intervention to preserve muscle strength and health [3].

## The social burden of Sarcopenia

**Fig. 3:**
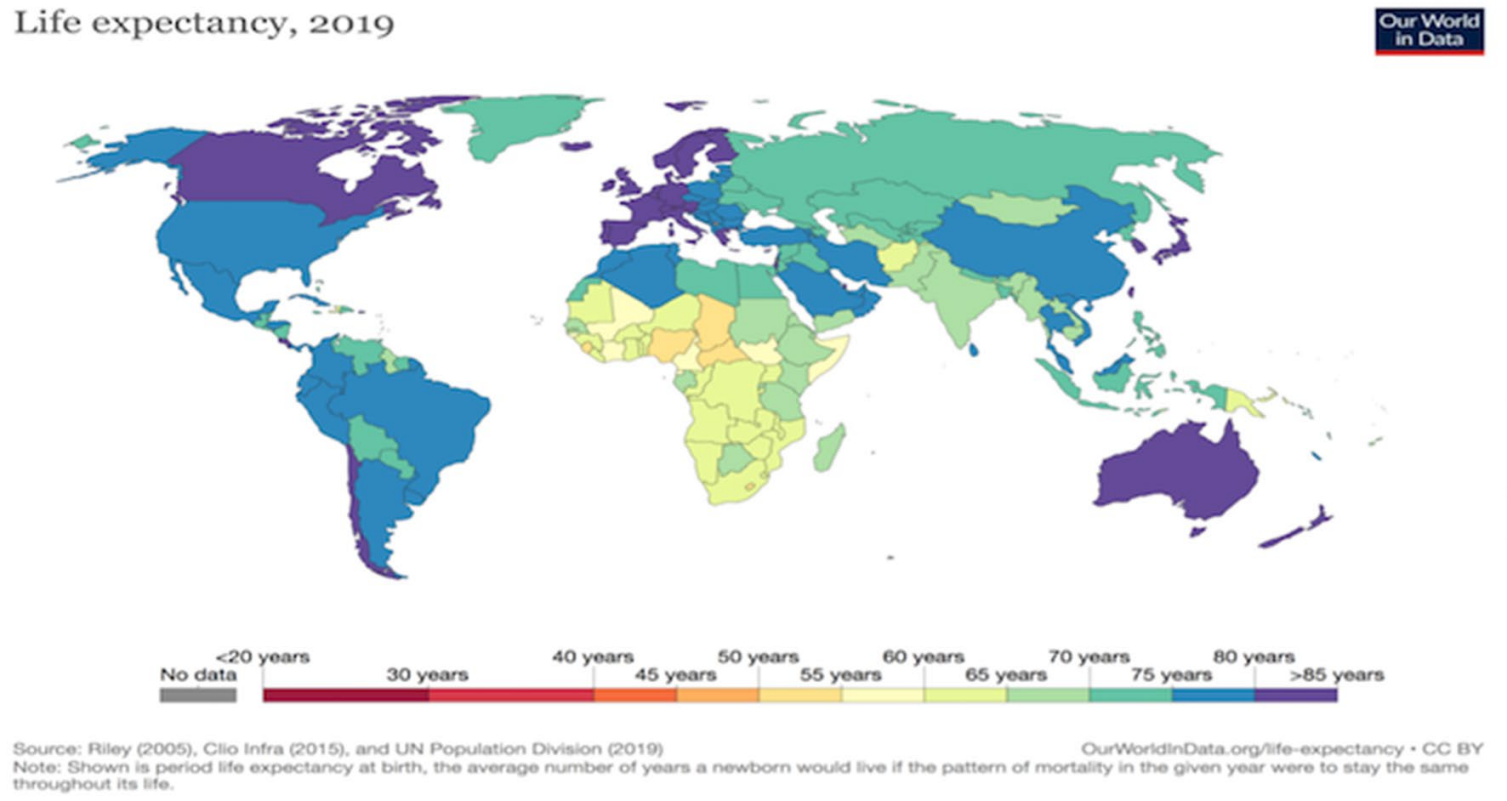
Latest data published by the United Nations for life expectancy in 2019

The social burden of sarcopenia is foreseen to increase, indeed world population older than 60 years of age is supposed increase in the next years as done in the last century (https://ourworldindata.org/life-expectancy) (Fig. 3). According to a systematic review report of the International Sarcopenia Initiative, prevalence of sarcopenia is likely to be 1–29% in community-dwelling populations, 14–33% in long-term care populations and 10% in the only acute hospital-care population [4, 7]. The economic implications of sarcopenia could impact on the health system [15]. Goates et al. [11] performed a new estimation on the economic load of sarcopenia using up-to-date sarcopenia classifications and hospital expenditure data. Goates et al. [11] found in 2019 that sarcopenia determined an average per person cost of USD $260 and globally USD $40.4 billion in the USA. Average per-person cost was different across ethnicity resulting higher for Hispanic women (USD $548) and lower for Non-Hispanic Black women (USD $25). Finally, patients affected by sarcopenia resulted to have greater odds of hospitalization (OR, 1.95; *p* < 0.001) compared to those without sarcopenia [11].

## Why measure sarcopenia using medical imaging?

**Fig. 4:**
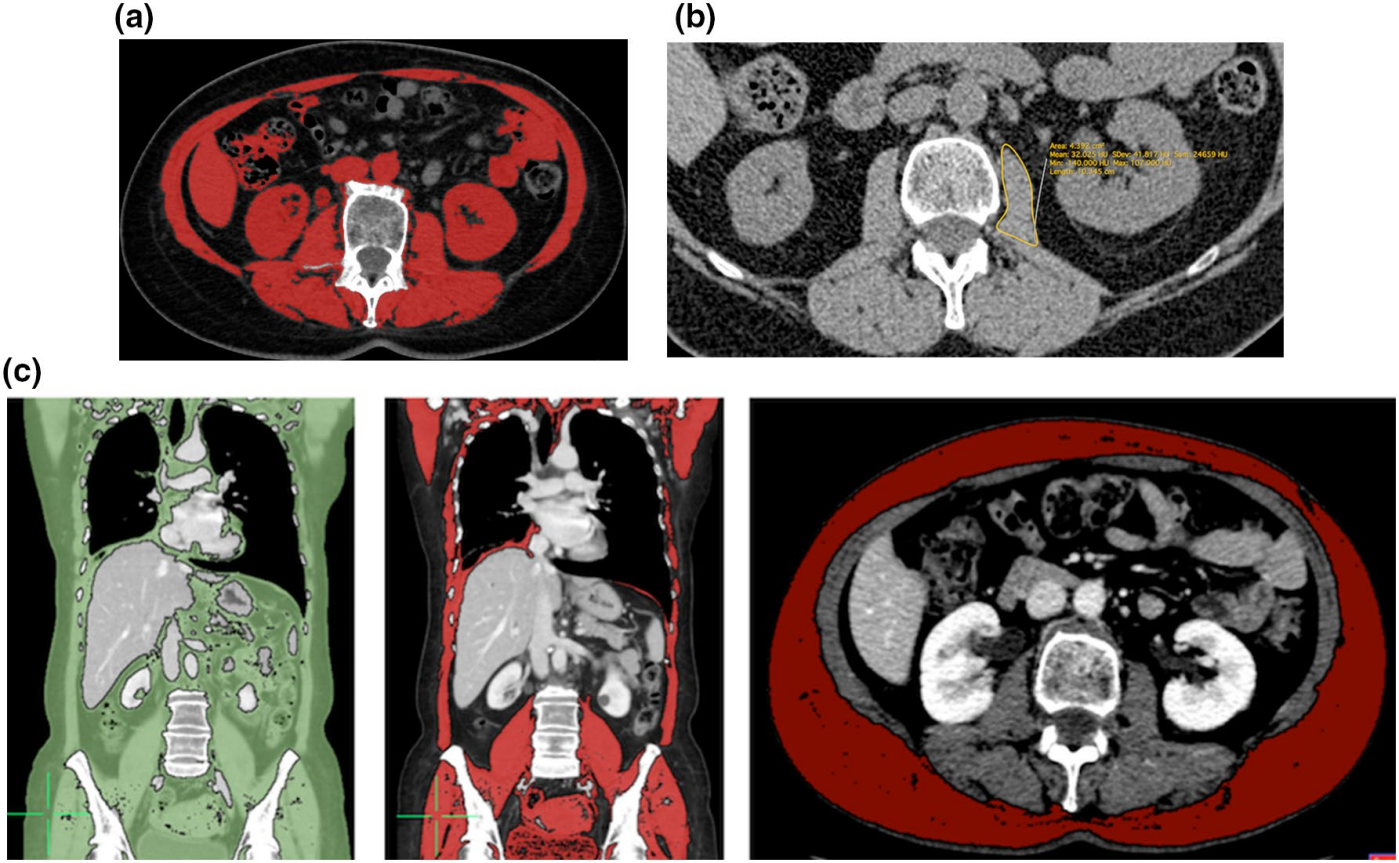
**a** Abdominal CT images at the third lumbar vertebra. Pitfalls are represented by incorrect segmentation of muscular tissue due to the fact that growing algorithms could be unable to distinguish muscular tissue from parts of the bowel and solid abdominal organs (kidney, major vessels and liver). Manual correction is essential. **b** Classical cross-sectional area evaluation at the L3 level showing muscle area and muscle density in Hounsfield unit. **c** Whole-body CT images taken with different planes showing some examples to estimate fatty tissue (green), muscular tissue (red), and subcutaneous fat (purple). Note that seeding method has been used; therefore, manual correction by an expert reader is mandatory

There are several reasons why radiologists should be aware that the topic “sarcopenia” has a clinically relevant role. The first is historical, indeed, with the almost complete left of osteoporosis evaluation, the radiological community lost the possibility to master the assessment of a useful clinical biomarker. By consequence, DXA is no more widely used in several radiological facilities and sometimes DXA is used in non-radiological environments. Therefore, the evaluation of sarcopenia is still an opportunity for the radiological community to master the assessment of a patient-specific imaging biomarker, able to predict several clinical outcomes. The second reason is that the amount of literature indicating that sarcopenia or muscle mass assessment on radiological techniques is an effective biomarker is rapidly increasing due to its clinical usefulness. In patient scheduled for oncological surgery, the presence of a reduced muscle mass or sarcopenia has been related to increased complications in the postoperative follow-up, increased days of hospitalization, lower tolerance to chemo- and radiotherapy and even mortality [12–19]. Reduced muscle mass and sarcopenia, not necessarily related to an impaired nutritional status, have been associated with worse outcome in patients with fractures, undergoing major surgery. Patients with low muscle mass have also an increased risk of falls and subsequent fractures [20–23]. Quantification of total muscle area with CT as a surrogate for muscle mass assessment has also been used in follow-up assessment in patients undergoing fasting mimicking diet and hormone therapy to induce breast cancer regression [24, 25]. In this complex study, muscle mass evaluation on CT was tailored on the clinical need even with innovative and original techniques (Fig. 4).

**Fig. 5:**
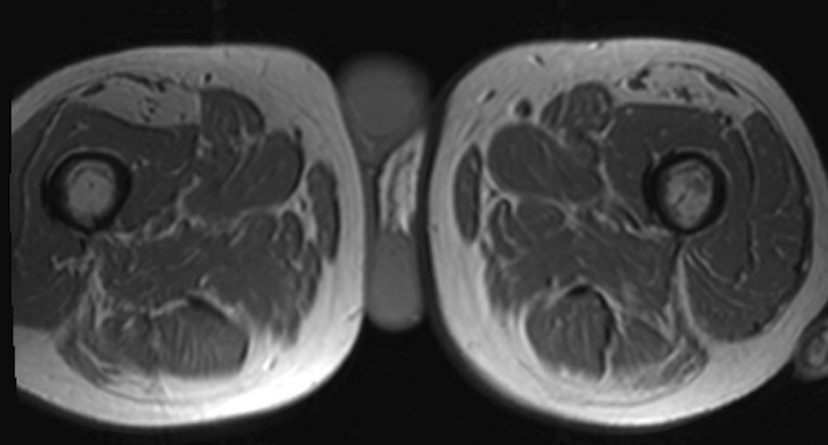
Vitamin D deficit in patients with selective atrophy of rectus femoris as shown by standard MRI of both thighs of an elderly man with 25-hydroxyvitamin D level of below 12 ng/mL

The third reason is that the detection of reduced muscle mass or sarcopenia is amenable of treatment with muscular training and nutritional supplementation (protein, amino acid, vitamin D and creatinine for example) to increase physical performance with muscle mass and strength augmentation [20, 26–31]. Regarding nutritional deficits, it has been evaluated that muscle impairment is associated with vitamin D deficiency [20, 31] and that there is an association between vitamin D deficiency and muscular atrophy and fatty infiltration visible with CT or MRI (Fig. 5) [31]. Indeed, diagnostic imaging can confirm that the skeletal muscle is a metabolic tissue responding not only to vitamin D, but also to IL-6, brain-derived neurotrophic factor, insulin, glucocorticoids, and thyroid hormones for example [29, 32]. Finally, the radiological community has the great chance to unveil and overcome the complexity of muscle mass and sarcopenia evaluation. Radiologist could become pivotal in suggesting when and how to use medical imaging to give reliable and clinically significant measures and cutoff points. Sarcopenia is clearly underdiagnosed in daily clinical practice, and radiologists have the chance to be inserted in the management of patients where muscle mass assessment is useful.

## Dual-energy X-ray absorptiometry

The EWGSOP recommended the use of dual-energy X-ray absorptiometry (DXA) to assess lean mass as an estimation of all non-fat/non-bone tissues, fat mass and bone mineral content [17]. Indeed, its use is suggested to calculate appendicular lean mass index (ALMI = ALM/height^2^) to define sarcopenia or low muscle mass using a defined cutoff of < 5.5 kg/m^2^ in women and ALMI < 7.0 kg/m^2^ [4]. The most relevant advantages of DXA are not only the use of a series of cutoff points to define the presence of sarcopenia, but also the relatively low radiation exposure (0.001 mSv approximately, less than a standard chest radiography). Moreover, DXA is cheaper compared to a standard CT scan and it is easy to be performed from a technical point of view. Unfortunately, DXA, even if accurately performed, has several limitations: low accuracy in estimating truncal fat and muscle due to the inability to separate intra-abdominal organs; over-/underestimation of the extent of sarcopenia or the presence of obesity from the amount of fat and muscle interpolated from arms and legs and low accuracy in the presence of edema and altered hydration status. Suboptimal patient positioning, lack of demographics reference data and un-experienced image analysis are typical pitfalls reducing DXA efficacy in clinical practice with possible implications for patients correct diagnostic classification and management [33]. Messina et al. [21] found that on 485 reports of DXA examinations, 451 (93%) had at least one error out of a total of 558 errors, most of which related to data analysis *n* = 441 (79%), followed by patient positioning *n* = 66 (12%) and demographics/artefacts. Among the disadvantages, DXA is not the optimal modality to estimate visceral fat, which is metabolically more active than subcutaneous fat and it is not sufficiently accurate to estimate thoracic and paraspinal muscles. Compared to CT and MRI, DXA cannot estimate muscle quality regarding fatty infiltration and the correlation with DXA-measured appendicular skeletal mass index (ASMI), adjusted by height, resulted to be only moderate if correlated with skeletal muscle index (SMI) based on CT (*r* = 0.41–0.66) [34]. Finally, DXA data resulted to predict the presence of muscle weakness but not mortality [35]. Despite these limitations, as suggested by EWGSOP, DXA is useful in clinical practice to confirm sarcopenia when there is a clinical suspicion and other reasons for low muscle strength have been excluded (such as depression, stroke, neurological balance disorders and peripheral vascular disorders) [6, 16, 17]. Indeed, in these patients, if low values or scores of muscle strength, grip strength and chair stand test are found, the use of radiological techniques is suggested mainly to confirm the presence of sarcopenia [6]. When using DXA, a basic index, which is the sum of the lean muscle mass of both arms and legs, called appendicular skeletal muscle mass (ASM), is usually adopted in clinical practice. ASM can be adjusted using the height squared (ASM/ht2), weight (ASM/wt), or BMI (ASM/BMI). Among these adjustments, the most frequently employed index is ASM/ht2 adopted in the EWGSOP guidelines and suggested to as Skeletal Muscle Index [35]. Several guidelines introduced the ASM/ht2 as a diagnostic index for sarcopenia (Table 1), but ASM/ht2 index in patients with high BMI, due to the large amount of fat, can be classified as non-sarcopenic.

**Table 1:** ASM/ht2 as a diagnostic index for sarcopenia

Guideline	Male	Female
EWGSOP 2010	ASM/ht2 ≤ 7.23 kg/m^2^	ASM/ht2 ≤ 5.67 kg/m^2^
IWGS 2011	ASM/ht2 ≤ 7.23 kg/m^2^	ASM/ht2 ≤ 5.67 kg/m^2^
AWGS 2014	ASM/ht2 ≤ 7.0 kg/m^2^	ASM/ht2 ≤ 5.4 kg/m^2^
FNIHSP 2014	ASM/BMI ≤ 0.789	ASM/BMI ≤ 0.512

*ASM* appendicular skeletal muscle, *AWGS* Asian Working Group for sarcopenia, *BMI* body mass index, *EWGSOP* European Working Group on sarcopenia in older people, *FNIHSP* Foundation for National Institutes of Health Sarcopenia Project, *IWGS* International Working Group on Sarcopenia

## Computed tomography

Computed tomography is routinely performed as a standard part of diagnostic investigations in both oncological and non-oncological settings. CT is also used as a screening tool to evaluate the presence of sarcopenia. Indeed, CT is probably the best technique to assess muscle mass and quality and it is considered the gold standard method of body composition analysis and diagnosis of abnormal body composition phenotypes, especially in nutritionally vulnerable patients [36].

There are several reasons to believe that CT is currently the best technique to assess body composition. The first reason is that CT is commonly used in different kinds of patients and its usage is going to increase through years. It has been estimated that in Italy where medical practice is generally derived by the public health system control and financing there was a general increase in the number of CT in an 11-year period for several reasons [37]. Italian data on CT usage increase are concordant with the increase in CT usage in the USA where the healthcare system is mainly private [Organization for Economic Co-operation and Development (OECD). Health care resources. OECD website.stats.oecd.org/index.aspx?queryid=30184. Accessed January 1, 2020]. The second reason is that CT usage will continue to increase not only because CT is a first-line diagnostic modality for various acute and chronic illnesses of aging, such as fractures and cancers, but also because there is more defensive medicine, increased CT screening for lung cancer and colon cancer, simplified methods to perform CT due to development of faster scanners and user-friendly interfaces. The third reason is that, using CT, it is possible to acquire quantitative tissue measurements in a highly reproducible way and some CT-derived data resulted to be strong associated with clinical outcomes [38, 39]. Unfortunately, CT has the significant disadvantage of radiation exposure; therefore, it is difficult to propose CT as a screening tool for sarcopenia in patients who do not need CT for other reasons. One of the simplest and fastest way to estimate whole-body skeletal muscle mass is to calculate the cross-sectional areas of the psoas muscle or of the abdominal muscle mass at the third (L3) or fourth (L4) lumbar vertebra because at these anatomical levels muscle mass should only marginally affected by movement [40]. Total muscles area at T4 level was also used [41]. On abdominal CT axial sections, the middle L3 level, it is possible to include the rectus abdominis, transverse abdominis, internal and external obliques, quadratus lumborum, psoas major and minor, and erector spinae. In this anatomical area, the trunk, DXA resulted to be limited; therefore, CT is essential [42]. A good correlation from CT-derived values obtained from a single CT slice and whole-body adipose tissue and skeletal muscle has been demonstrated [43]. A threshold range of 29 to 150 HU is commonly used to define the muscle, whereas adipose tissue typically ranges from − 30 to − 190 Hounsfield units. In addition, using CT it is possible to assess the presence of intramuscular fat in two ways: directly identifying fatty areas inside the muscle or showing decreased HU attenuation due to myosteatosis (low muscle density based on average muscle density). Myosteatosis or intramuscular adipose tissue content (IMAC) can be obtained with a simple formula: IMAC = CT attenuation value of the multifidus muscles divided by CT attenuation value of subcutaneous fat [44]. CT wider usage is limited by the fact that an 8 mSv radiation dose typical of an abdomen/pelvis CT (2.5 mSv for the natural environment) is not acceptable for sarcopenia-related screening purposes in healthy subjects. Another limitation of CT is that intra-myocellular fat and intermuscular fat cannot be differentiated, whereas using MRI it is possible a more accurate quantitative estimation of intramuscular fat. CT measurement can be done manually drawing regions of interest (ROIs) using standardized thresholds on non-contrast-enhanced images because tissue enhancement affects muscle attenuation values. Muscular CSA has to be adjusted regarding height (CSA/height^2^) to estimate the skeletal muscle index (SMI). Common SMI cutoff values range from 52 to 55 cm^2^/m^2^ for men and from 39 to 41 cm^2^/m^2^ for women according to a recent meta-analysis [45]. Sarcopenia on CT can be also calculated using specific automatic software, some of them are free and public domain (NIH-ImageJ) [46]. When manually positioning ROIs or when manually tracing the relevant muscular abdominal regions, care should be taken to avoid common pitfalls. Typical pitfalls are represented by incorrect segmentation of adipose tissue due to the fact that growing algorithms could be unable to distinguish VAT from subcutaneous adipose tissue and inclusion of parts of the bowel as VAT (Fig. 4). These errors require manual correction by an expert reader [47]. If no semi- or fully automated segmentation methods are used, manual segmentations are still time-consuming and disturbed by interobserver variability (Fig. 4). Most studies used sarcopenia cutoff points defined as Skeletal Muscle Index (SMI) generally < 41 cm^2^/m^2^ or < 38.5 cm^2^/m^2^ [48], but a solid and evidence-based consensus on standardized CT thresholds to diagnose sarcopenia is not present at the moment.

## Magnetic resonance imaging

**Fig. 6:**
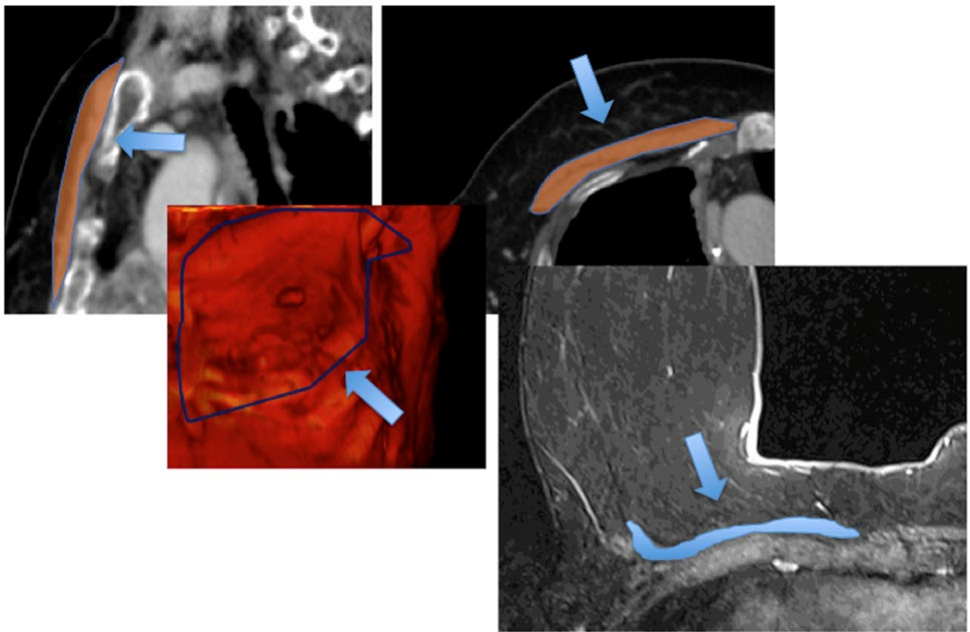
Thoracic CT and MRI images taken at the Lewis angle to estimate muscle mass on pectoralis muscle (arrows). Examples from patent number: 102019000016049

The different numbers of protons inside muscular and fatty tissue guarantee a high contrast resolution and accuracy to assess muscle CSA in different anatomical areas with a very high correlation with CT data with old and new equipment [48–51]. MRI has been used mainly in research settings; therefore, it is difficult to find established and approved imaging protocols, reference data and cutoffs. MRI adds new information regarding muscular status detecting intra-muscular edema, fibrous substitution and even muscular elasticity and contraction. Several well-known MRI sequences are able to separate easily fat from water, such as the three-point Dixon technique and spectroscopy. Other MRI sequences have been used in several research settings, such as strain rate tensor imaging, diffusion tensor imaging T2-mapping, and multiecho sequences used for T2 mapping with limited clinical impact [52]. One clear advantage of MRI is that it is radiation-free technique. On the contrary, the doubtful clinical advantage of MRI over other well-established techniques as well as no protocol standardization, high costs and difficult post-processing limit wide MRI usage in sarcopenia assessment. Recently, MRI has been used as a CT surrogate to estimate muscular mass in patients affected by breast cancer who underwent breast MRI for breast cancer evaluation. Rossi et al. [50, 51] found that pectoralis muscle area on breast MRI could be used to estimate muscle mass in women with breast cancer (Fig. 6); therefore, pectoralis muscle area assessment could be a potential marker of muscular status in women with breast MRI available. Indeed, high-risk women or early breast cancer patients could have no standard body CT available and breast MRI could directly define muscular status and influence patient personalized therapies. MRI can be used in alternative to CT because the segmentation of muscles on cross-sectional images is strongly correlated and coefficients up to *r* = 0.99 have been obtained when using cadavers as references [49]. MRI can give overlapping information as CT regarding muscle mass cross-sectional area, volume and even fatty infiltration. Fatty infiltration on MRI can be assessed using multiecho gradient echo sequences to quantify intramuscular fat [53]. Using different MRI applications and sequences, it is also possible, mainly in research settings, to estimate intra-muscular edema, which can be shown using fluid-sensitive sequences, fibrous or scarring infiltrations and residual muscular contractility and elasticity. [54] For these advanced MRI applications, Dixon-based MRI sequences resulted to be more reliable and easier to be handled than spectroscopy Dixon [53]. Both single-voxel and multi-voxel spectroscopy, the latter with the goal to overcome the irregular pattern of muscle fatty or scar infiltration, have been reported for sarcopenia assessment, but [55–57] with difficult implementation in clinical practice. Muscular contractility and elasticity have been studied using MRI [58] with sequences adopting strain rate tensor measures detecting the principal directions and magnitude of the instantaneous deformation providing information on the alignment of muscular fibers orientation and deformation in the plane perpendicular to the muscle long axis. The strain rate orientation and deformation could give information on muscle fiber arrangement as well as information on the extracellular matrix, which is the non-contractile muscular tissue [58]. Mapping of muscular fibers using strain rate tensor MRI was used to study the skeletal muscle in different ages and muscular regional properties during motion to find differences in contractility and elastic properties. Other MRI sequences such as diffusion tensor imaging and multiecho sequences used for T2 mapping have been used showing differences between normal and sarcopenic muscles, especially in the elderly, but these applications are still limited for research application [52]. MRI will be likely introduced in clinical practice when issues related to protocol standardization, costs, acquisition and post-processing time will be resolved.

## Ultrasound

Clinical ultrasound (US) is a widely used practical diagnostic tool with known advantages, such as easiness of use, repeatability, lack of ionizing radiation, portability and availability. In several clinical settings, especially when it is difficult to obtain CT or MRI, US can be considered the best tool to evaluate patients directly at the bedside or even at home with good intra- and interobserver agreement. US can be used to evaluate both muscle quantity and quality. Recently, the European Geriatric Medicine Society suggested a protocol for US usage in muscle mass assessments [REF].

Clinical radiologists will have the opportunity to introduce muscular assessment in daily clinical practice enhancing the role of US in the prevention of sarcopenia-related disorders. Muscle thickness (MT), cross-sectional area (CSA), echo intensity (EI), pennation angle (PA), fascicle length (FL), physiologic cross-sectional area (PCSA), contrast-enhanced assessment of vascularization and elastography are the main parameters amenable of US-based evaluation.

**Fig. 7:**
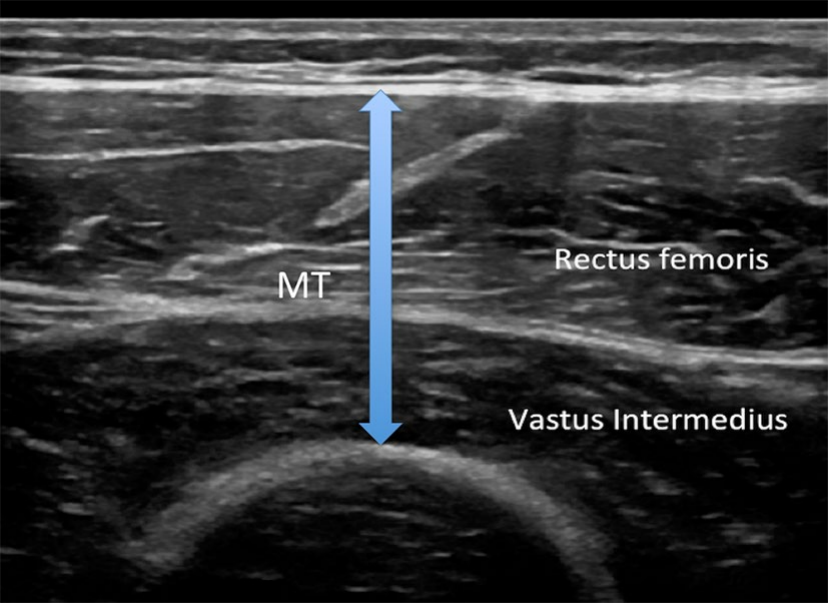
Evaluation of the anterior compartment of the thigh could be considered a good anatomical area to take US-derived measurements; in this case, the rectus femoris and the vastus intermedius are shown. Muscular thickness (MT) is measured as a distance between the superficial aponeurosis and femur including the rectus femoris and vastus intermedius muscles. Cross-sectional area of the rectus femoris muscle can be measured from transversal US images drawing a region of interest (ROI) using either a freehand or a polygon tool. ROI should include most of the rectus femoris, excluding the muscle fascia

MT or CSA of the muscle correlates can be used to confirm the presence of muscle mass depletion and correlates with DXA, CT and MRI measurements [22]. It has to be remembered that the site-specific muscle loss, diagnosed by US, a phenomenon known as “regional” or “site-specific” sarcopenia had low correlation with functional parameters that should always incorporated in sarcopenia assessment. Considering that sarcopenia is site specific and that muscle loss is greater in the lower than the upper limbs, evaluation of the anterior compartment of the thigh could be considered a good anatomical area to take US-derived measurements (Fig. 7). The multiple advantages of US usage in sarcopenia evaluation are counterbalanced by the actual lack of normative data on a population-based scale sarcopenia, the lack in standard protocols and cutoff points for US-based diagnosis of sarcopenia.

## Conclusion

The continuous growing number of patients undergoing different kinds of radiological examinations is a greedy opportunity for radiologists to include muscle mass quantitative evaluation in the routine examinations of different kinds of patients. Using CT, MRI and even US, it is possible to monitor muscle changes in size and architecture. With radiological techniques, it would be possible to identify patients at risk for sarcopenia-related morbidities and suggest preventive interventions.

Radiologists can be pivotal in muscle mass and sarcopenia assessment and can strongly influence patient care. Radiologist should not miss this opportunity as already done in the past with other pathological conditions (e.g., osteoporosis). Finally, sarcopenia evaluation with radiological methods could enhance the role of radiologists in performing studies with relevant impact on medical and social outcome. This is a good chance to place radiology at the pinnacle of quality in evidence-based practice with high-level studies [59].
